# Cryptococcal Osteomyelitis in the Ribs

**DOI:** 10.4103/0974-777X.59253

**Published:** 2010

**Authors:** Somika Sethi

**Affiliations:** *Department of Pathology, Senior Resident, Sir Ganga Ram Hospital, Subimal Roy, Pathology, Senior Consultant, Sir Ganga Ram Hospital New Delhi - 110 060, India*

**Keywords:** Cryptococcus, Osteomyelitis, Immunocompetent

## Abstract

Isolated cryptococcal osteomyelitis, in an immunocompetent, is rare and only a few cases have been reported in literature. We present the case of a 30-year-old man presented with pain on the left side of chest with fever and gradually increasing swelling in left lateral lower aspect of chest. Investigation revealed a lytic lesion in the anterior end of left 6^th^ rib with normal CD4 count. He was tested negative for HIV antigen. Excision of the sixth rib, morphologically revealed cryptococcal osteomyelitis and the patient was given anti-fungal treatment for six months.

## INTRODUCTION

Bone involvement occurs in 5-10% of reported cases of disseminated infection with Cryptococcus neoformans. However, isolated cryptococcal osteomyelitis is a rare entity, the vertebral column being the most frequent site of involvement.[1] We would like to share a case of cyrptococcal osteomyelitis involving the left sixth rib that we recently came across in our practice, because of its unique presentation in an immunocompetent patient.

## CASE REPORT

A 30-year-old non-diabetic, non-hypertensive man developed pain in left side of the chest, pleuritic in nature and localized to left lateral aspect not associated with breathlessness and cough. Subsequently, he developed fever not associated with chills and rigor and swelling on the left lateral lower aspect of the chest, which was painful and gradually increasing. X-ray and CT Scan revealed a lytic leision in the anterior end of left sixth rib with associated subcutaneous soft tissue swelling and enlarged mediatsinal lymphnodes. The patient was tested for HIV antigen and was negative. On the left lower aspect of chest, the patient showed a tender swelling which measured 6 × 5 cms. His haemoglobin and total leucocyte count were normal. Patient's CD4 count was normal and Mantoux test was positive, but Quantiferon gold was negative. Computed Tomography (CT) of head was also done, which was normal. Fine needle aspiration cytology (FNAC) was performed outside and suggestive of cryptoccocal infection. The patient was taken for surgery and 6^th^ rib excision was done.

**Figure 1:**
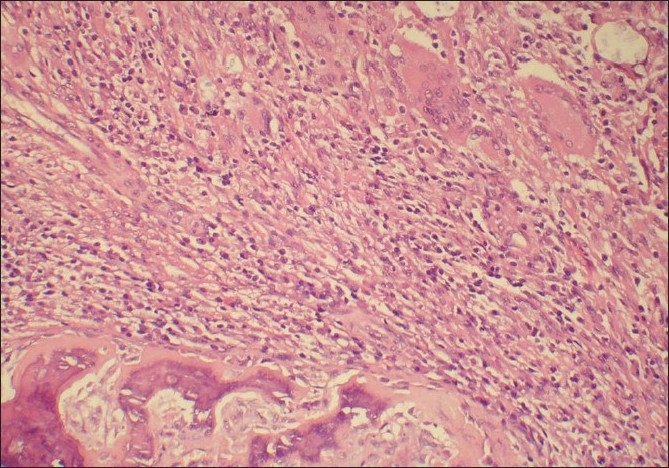
Bony lamellae surrounded by florid granulomatous inflammation (H and E, ×100)

**Figure 2:**
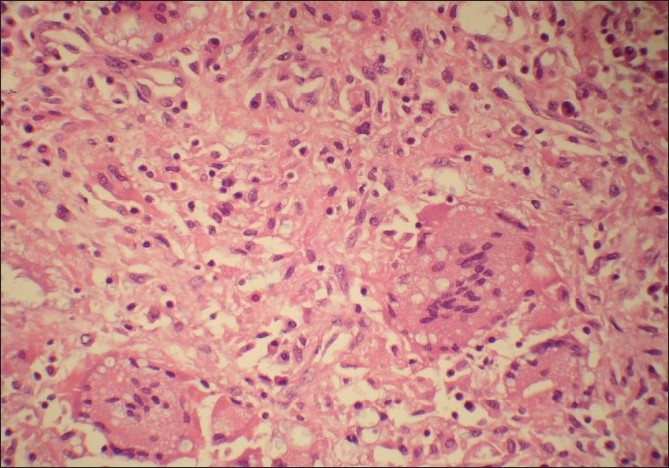
Intracellular inclusions within the giant cells (H and E, ×400)

Histopatholoical examination showed fragments of partly necrotic bone and diffuse chronic inflammatory cell infiltrate including many giant cells, histiocytes, lymphocytes and plasma cells [Figure 1]. The inflammation extended into the subcutaneous soft tissue forming microabcessess. Numerous encapsulated yeast forms, a few showing budding were present within the giant cells [Figure 2] and extracellulary within florid granulomatous inflammation [Figure 2].

**Figure 3:**
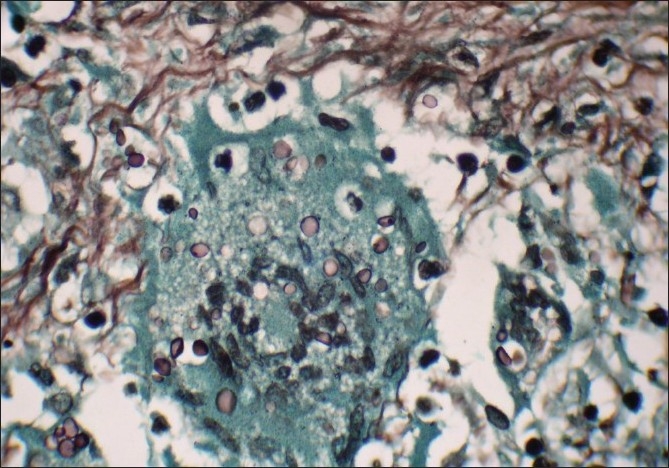
Intracellular inclusions, few showing narrow base budding (Gomori methamine silver stain, ×1000)

**Figure 4:**
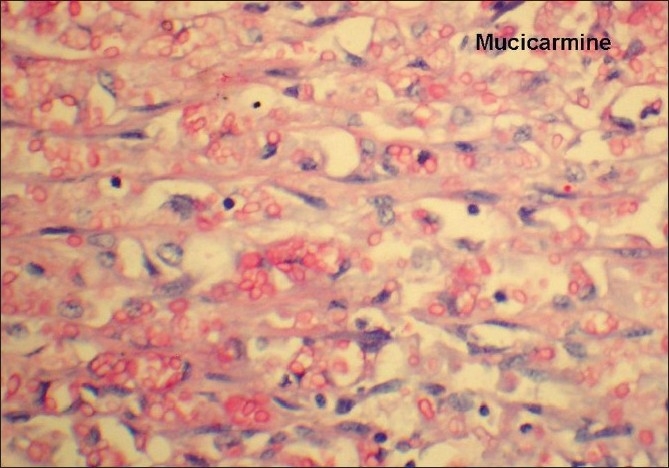
Mucicarmine stain highlighting the extracellular fungal spores (H and E, ×400)

A Gomori methenamine silver stain and mucicarmine stain highlighted yeast formed with narrow based budding [Figures 3 and 4]. A final diagnosis of cryptococcal osteomyelitis was given. Culture done for tuberculosis and sputum sent for acid fast bacilli were negative. The patient was started on antifungal treatment and was doing well in his last follow-up.

## DISCUSSION

Cryptococcosis is usually disseminated infection of individual hosts caused by inhalation of Cryptococcus neoformans.[2] It commonly occurs in immunocompromised individuals, particularly those with defective cellular immunity. The most commonly involved sites are lungs and CNS. Isolated bone involvement is very uncommon in an immunocompetent host. A review of 40 cases of cryptococcal osteomyelitis, by Liu,[3] reported sarcoidosis as the most frequent underlying disorder, followed by tuberculosis, steroid therapy and diabetes mellitus. Majority of the patients with skeletal cryptococcal infection presented with soft tissue swelling and tenderness. Seven out of 39 cases were febrile and the median duration of symptoms before diagnosis was three months, according to a study by Behrman *et al*.[4] The insidious course of the disease may lead to delay in diagnosis and the treatment management of isolated cryptococcal osteomyelitis is controversial because of its rarity. However, in the present case, surgery was done to minimize the chance of CNS involvement and antifungal antibiotics were administered for six months. The patient was doing well on his last follow-up.

## CONCLUSION

Cryptococcal skeletal infections can lead to significant morbidity and mortality and should be considered in differential diagnosis of lytic osseus lesions, even in immunocompetent patients.
